# Differentiating benign from malignant pulmonary nodules in the context of bronchiectasis: a retrospective study

**DOI:** 10.1080/07853890.2026.2681234

**Published:** 2026-06-09

**Authors:** Lin Wang, Danhui Yang, Xianglin Zhou, Ting Guo, Lv Liu, Cheng Lei, Hong Luo

**Affiliations:** ^a^ Department of Pulmonary and Critical Care Medicine, the Second Xiangya Hospital, Central South University, Changsha, Hunan, China; ^b^ Research Unit of Respiratory Disease, Central South University, Changsha, Hunan, China; ^c^ Clinical Medical Research Center for Pulmonary and Critical Care Medicine in Hunan Province, Changsha, Hunan, China; ^d^ Diagnosis and Treatment Center of Respiratory Disease in Hunan Province, Changsha, Hunan, China; ^e^ Department of Pathology, The Second Xiangya Hospital, Central South University, Changsha, Hunan, China

**Keywords:** Bronchiectasis, pulmonary nodule, benign nodules, malignant nodules, risk factors

## Abstract

**Background:**

Pulmonary nodules are common on chest CT in bronchiectasis (BE) patients. This study identifies risk factors for nodule malignancy in BE patients with pulmonary nodules.

**Method:**

We screened BE patients who underwent chest CT at Second Xiangya Hospital from Jan 1, 2019 to Mar 31, 2025. Only patients with pulmonary nodules and pathological results were enrolled. Univariate and multivariate logistic regression were performed to identify independent predictors of malignancy.

**Results:**

, Of 143 patients, 28 had benign nodules and 115 had malignant nodules. Malignant nodules were more often pure ground‑glass/ground glass with a solid component nodule type (73.9% vs 28.6%, p<0.001) and upper lobe located (54.8% vs 32.1%, p=0.032). Benign nodules most commonly showed inflammation (60.7%), while malignant nodules were predominantly invasive adenocarcinoma (86.9%). Pure ground‑glass/ground glass with a solid component nodule type was the only independent predictor of malignancy (adjusted OR 8.53, 95% CI 3.14–25.81). Upper lobe location did not remain significant after adjustment. Reiff score and same‑lobe BE‑nodule coexistence were not significantly associated.

**Conclusion:**

Among BE patients with pulmonary nodules, pure ground‑glass/ground glass with a solid component nodule type independently predicts malignancy. This may aid clinical decision‑making.

## Background

Bronchiectasis (BE) is a chronic respiratory disease characterized by irreversible bronchial dilation, often accompanied by impaired mucociliary clearance, chronic airway inflammation, and bacterial colonization [1]. With the widespread use of high-resolution computed tomography (CT), the co-existence of bronchiectasis with other pulmonary diseases detected on high-resolution CT has been increasingly recognized.

Pulmonary nodules are defined as lung opacities that measure less than 3 cm in diameter and are surrounded by lung parenchyma [2]. According to data from the United States, the incidence of incidental pulmonary nodules is 5.8 per 100,000 person-years in women and 5.2 per 100,000 person-years in men [2]. However, although approximately 90% of pulmonary nodules are benign, the uncertainty in determining their likelihood of malignancy leads to a series of problems. These include excessive diagnostic evaluations such as repeated high-resolution CT scans, nonsurgical biopsy or surgical resection and even potential overtreatment of benign nodules [3]. These excessive evaluations not only increase the healthcare costs but may also cause complications, increase patient anxiety, impair quality of life, and even lead to premature death [4]. Therefore, accurately assessing the probability of nodules malignancy is essential for clinical practice.

Although both bronchiectasis and pulmonary nodules have been extensively clarified, the presence of chronic airway inflammation and structural lung damage in bronchiectasis may alter the natural history of pulmonary nodules and confound traditional risk prediction models. To date, no specific risk stratification tools exist for this distinct population. Our study retrospectively analyzed clinical, radiological and pathological data of pulmonary nodules in BE with pulmonary nodules patients. The purpose of this study was to identify risk factors associated with nodule malignancy in this population.

## Methods

**Figure 1.**
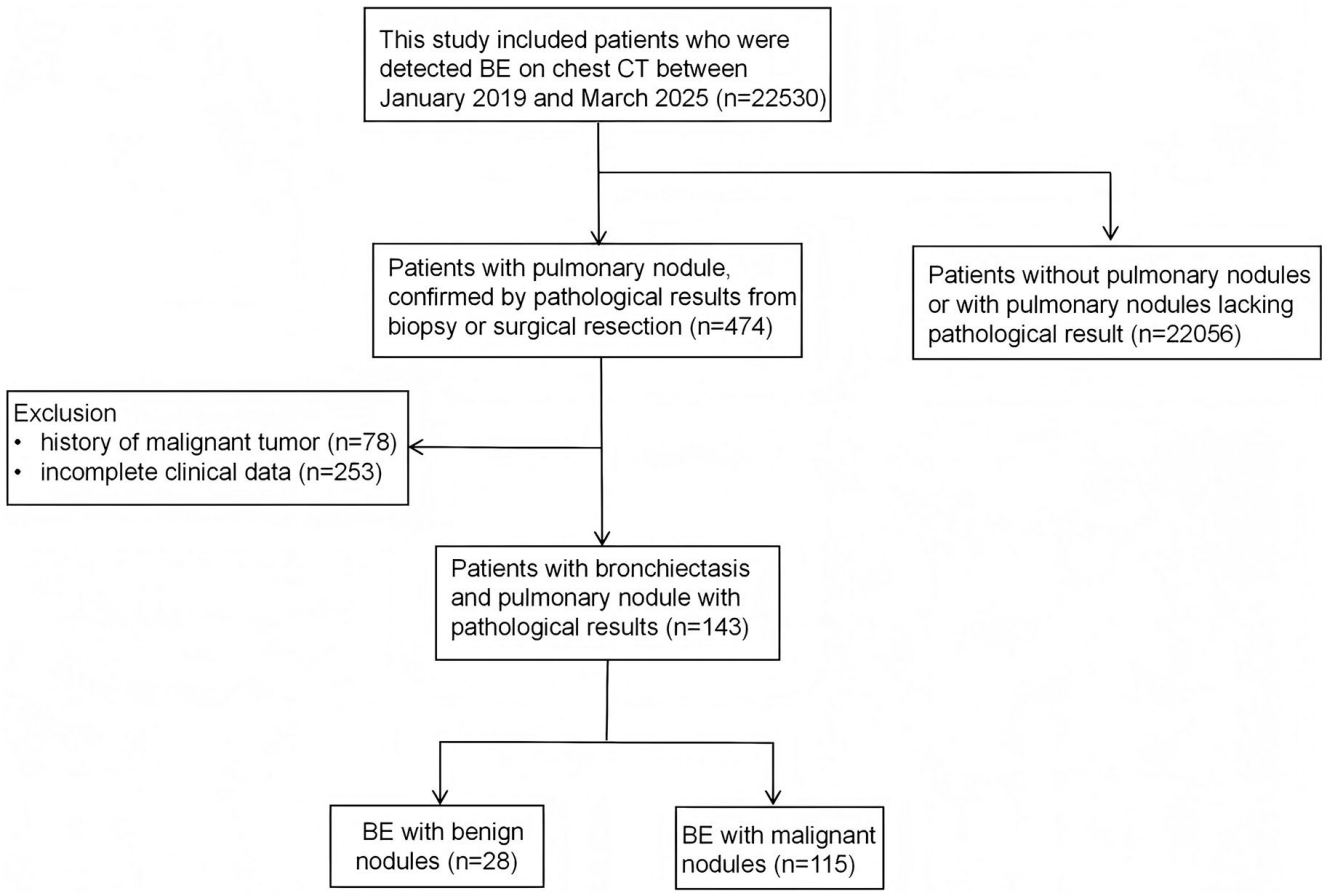
Flowchart summarizing patient selection in this study.

This study was designed as a single-center, retrospective and observational cohort study. Specifically, the study population included adult patients (>18 years old) from January 1, 2019 to March 30, 2025 at the Second Xiangya Hospital of Central South University, who had bronchiectasis and pulmonary nodules identified on chest CT scans and whose pulmonary nodules had pathological results by biopsy or surgical resection. Patients with a history of malignant tumor or incomplete medical records (medical history) were excluded from the main clinical analysis. In addition, we conducted descriptive analyses of radiological and pathological features in all patients with pathologically confirmed pulmonary nodules, regardless of clinical data completeness, and these results are provided as (supplementary material Table S1 and Figure S1). The flow diagram enrolling the patients for analysis is shown in Figure 1. Among patients with multiple pulmonary nodules, the nodule that was analyzed was the one subjected to biopsy or surgical resection. Based on pathological results, all enrolled patients were divided into two groups: BE with benign nodules and BE with malignant nodules. This study was approved by the Second Xiangya Hospital of Central South University (LYEC2025-K0246). The clinical research ethics committee of the Second Xiangya Hospital, Central South University waived the requirement for obtaining written informed consent. Before data analysis, patient records were anonymized and de-identified.

The following data were retrospectively collected for eligible patients including gender, age, height, weight, smoking history, comorbidities, history of tuberculosis, spirometry measurements, size, type, edge features (including irregular, lobulated, or spiculated edges), the number and location of affected lobe of pulmonary nodules [5], pathological results and genetic test results for malignant nodules. Nodule type was classified according to the Fleischner Society terminology: pure ground-glass nodule (pGGN), part-solid nodule (mGGN: ground-glass with a solid component), and solid nodule.

All chest CT images were independently reviewed by one radiologist and one pulmonologist, both of whom were blinded to the pathological results of the pulmonary nodules. The lung was divided into six lobes (right upper lobe, right middle lobe, right lower lobe, left upper lobe, left lingular lobe and left lower lobe) for assessing the location of nodule and bronchiectasis. Radiological severity of bronchiectasis was assessed using a modified Reiff score. This score integrates the number of involved lobes (maximum of 6, with the lingular lobe counted separately) and the severity of bronchiectasis (cylindrical = 1, varicose = 2 and cystic = 3) [6]. The total score ranges from 1 to 18. Among patients with coexisting emphysema, the severity of emphysema was assessed visually using the modified Goddard scoring system. Each lobe was scored based on the extent of involvement (normal = 0; ≤5% = 0.5; ≤25% = 1; ≤50% = 2; ≤75% = 3; and >75% = 4). The average score of the six lobes was calculated as the patient’s emphysema severity score. Finally, patients were categorized accordingly into three groups: none/mild (average score <1); moderate (1≤ average severity score <2.5); and severe (average score ≥2.5) [7,8].

Continuous variables were presented as medians and interquartile ranges, and group comparisons were performed using Wilcoxon rank-sum tests. Categorical variables were reported as counts and frequencies and group comparisons were made using the chi-square test or Fisher’s exact test. To identify independent risk predictors for nodule malignancy, univariate logistic regression analysis was first performed. Variables with *p* < 0.10 in univariate analysis (upper lobe location, nodule type) were entered into multivariate logistic regression. Additionally, clinically relevant covariates including age, smoking history, COPD, nodule size, and edge features were forced into the model to adjust for potential confounding. Results are presented as adjusted odds ratios (aOR) with 95% confidence intervals. Furthermore, we constructed five hierarchical multivariable logistic regression models to assess the association between nodule malignancy and two factors: the Reiff score and the coexistence of BE and pulmonary nodule in the same lobe: model 1 adjusted for age and gender, model 2 additionally adjusted for smoking history, model 3 further adjusted for location of the nodule in the upper lobe beyond model 2, model 4 further adjusted for pure ground glass/ground glass with a solid component nodule type beyond model 3 and model 5 further adjusted for edge features beyond model 4. All statistical analyses were performed using the R language (R version 4.5.1), with P-value < 0.05 considered statistically significant.

## Results

Among 474 BE patients with pulmonary nodules and definitive pathological results identified between January 1, 2019 and March 30, 2025, 253 (53.4%) were excluded due to incomplete clinical data, and 78 patients were excluded due to a history of malignant tumors, resulting in 143 patients (30.2%) with complete clinical information included in the main analysis.

Among the 143 patients with complete clinical data, no significant differences were observed between benign and malignant nodule groups regarding age, BMI, smoking history, spirometry measurements, emphysema severity, the Reiff score, the coexistence of BE and pulmonary nodule in the same lung lobe, and the number of single or multiple nodules (Table 1). The median nodule size was 17.4 mm (IQR 12.6–21.1) for benign nodules and 16.3 mm (IQR 12.0–21.4) for malignant nodules. Edge features were observed in 60.7% of benign nodules and 56.5% of malignant nodules. The proportion of pure ground-glass and ground glass with a solid component nodule type was 73.9% in malignant nodules versus 28.6% in benign nodules (*p* < 0.001), and the proportion of upper-lobe nodules was 54.8% versus 32.1% (*p* = 0.032) (Table 2).

**Table 1.** Comparison of clinical data between BE patients with benign nodules and malignant nodules.

	BE with benign nodules (*n* = 28)	BE with malignant nodules (*n* = 115)	*P*	
Age	59.5(55.5, 66.2)	59(55, 67)	0.957	
Gender, n(%)			0.585	
Male	15(53.6)	55(47.8)		
Female	13(46.4)	60(52.2)		
BMI, median(IQR)	23.2(22.4, 25.3)	23.7(21.3, 26.3)	0.903	
Smoking history, n(%)			0.857	
Never	18(64.3)	76(66.1)		
Former/Current	10(35.7)	39(33.9)		
History of tuberculosis, n(%)	3(10.7)	4(3.5)	0.136	
Comorbidity, n(%)				
COPD	3(10.7)	14(12.2)	1.000	
Asthma	0(0.0)	3(2.6)	1.000	
Hypertension	7(25.0)	29(25.2)	0.981	
Diabetes	5(17.8)	10(8.7)	0.173	
Comorbidity Score*, median(IQR)	2(0, 4)	2(0, 4)		0.900
Spirometry, median(IQR)				
FEV1/FVC	78.4(75.2, 82.6)	80.0(74.6, 87.0)		0.500
FEV1 (L)	2.01(1.96, 2.39)	2.28(1.94, 2.64)		0.098
FEV1 predicted (%)	90.2(80.0, 99.8)	97.4(83.8, 109.0)		0.112
Presence and severity of emphysema, n(%)				0.874
None/Mild	26(92.8)	104(90.4)		
Moderate	1(3.6)	7(6.1)		
Severe	1(3.6)	4(3.5)		
Nodule number, n(%)				1.000
Single nodule	4(14.3)	18(15.7)		
Multiple nodules	24(85.7)	97(84.3)		
Total Reiff scores, median(IQR)	5(3, 6)	4(3, 6)		0.405
The positional relationship between bronchiectasis and pulmonary nodules, n(%)				0.634
Bronchiectasis and pulmonary nodules were located in the same lung lobe	21(75.0)	91(79.1)		
Bronchiectasis and pulmonary nodules were not located in the same lung lobe	7(25.0)	24(20.9)		

Data are median (IQR) or n (%). FEV1: forced expiratory volume in one second; FVC: forced vital capacity.*The Comorbidity Score was calculated using the BACI scoring system: metastatic malignancy (12 points), hematological malignancy (6 points), COPD (5 points), cognitive impairment (5 points), inflammatory bowel disease (4 points), liver disease (4 points), connective tissue disease (3 points), iron deficiency anemia (3 points), diabetes mellitus (3 points), asthma (3 points), pulmonary hypertension (3 points), peripheral vascular disease (2 points), ischemic heart disease/coronary artery disease (2 points), For each comorbidity presented in a patient, the corresponding points are assigned, and the total score is calculated by summing the points for all present comorbidities.

**Table 2.** Comparison of nodule radiological features between BE patients with benign nodules and malignant nodules.

	BE with benign nodules (n = 28)	BE with malignant nodules (n = 115)	*P*
Nodule size, median(IQR)	17.4(12.6, 21.1)	16.3(12, 21.4)	0.980
Upper lobes^1^, n(%)	9(32.1)	63(54.8)	0.032
Nodule type			<0.001
Subsolid nodules (pGGN^2^ or mGGN^3^), n(%)	8(28.6)	85(73.9)	
solid, n(%)	20(71.4)	30(26.1)	
Edge features^4^, n(%)	17(60.7)	65(56.5)	0.687

^1^Upper lobes mean nodule located in upper lobe.

^2^pGGN means pure ground glass nodule.

^3^mGGN means mixed ground glass nodule (ground glass with a solid component nodule).

^4^Edge features mean irregular, lobulated, or spiculated edges.

**Figure 2.**
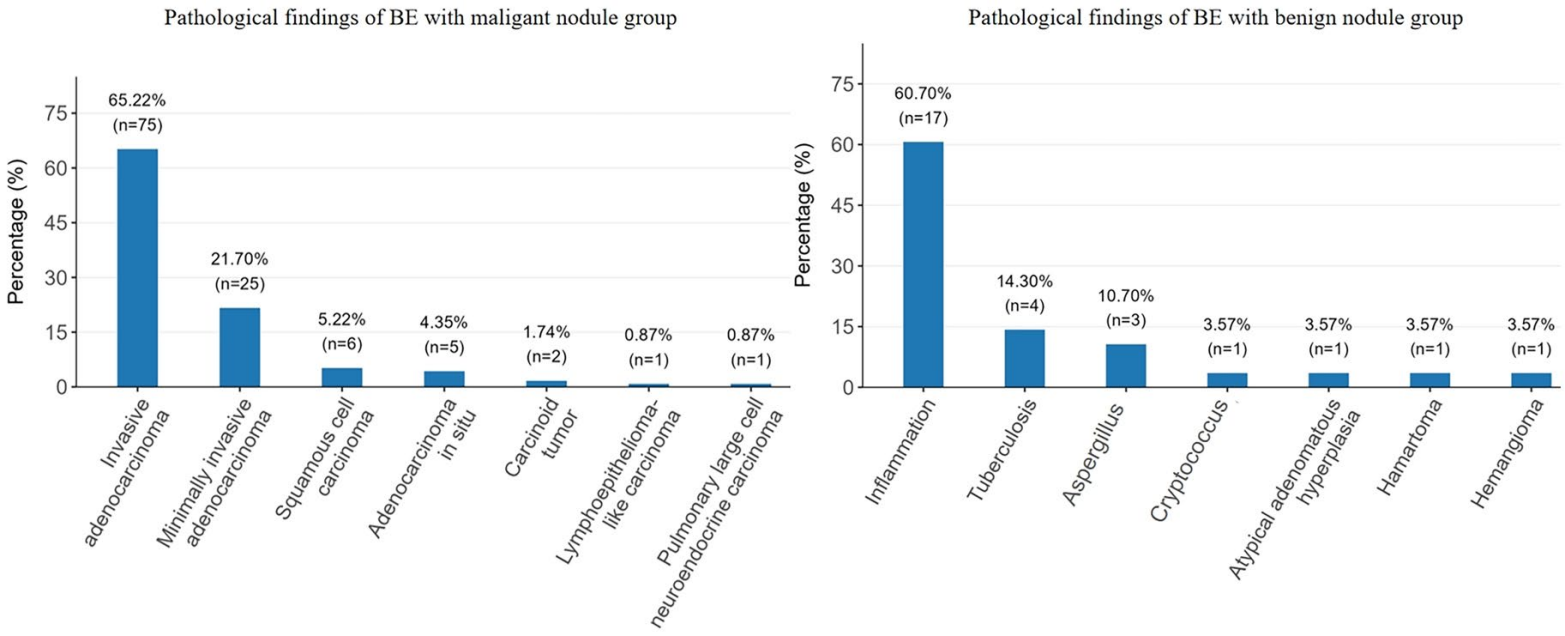
The pathological findings of BE patients with benign nodules and malignant nodules.

**Figure 3.**
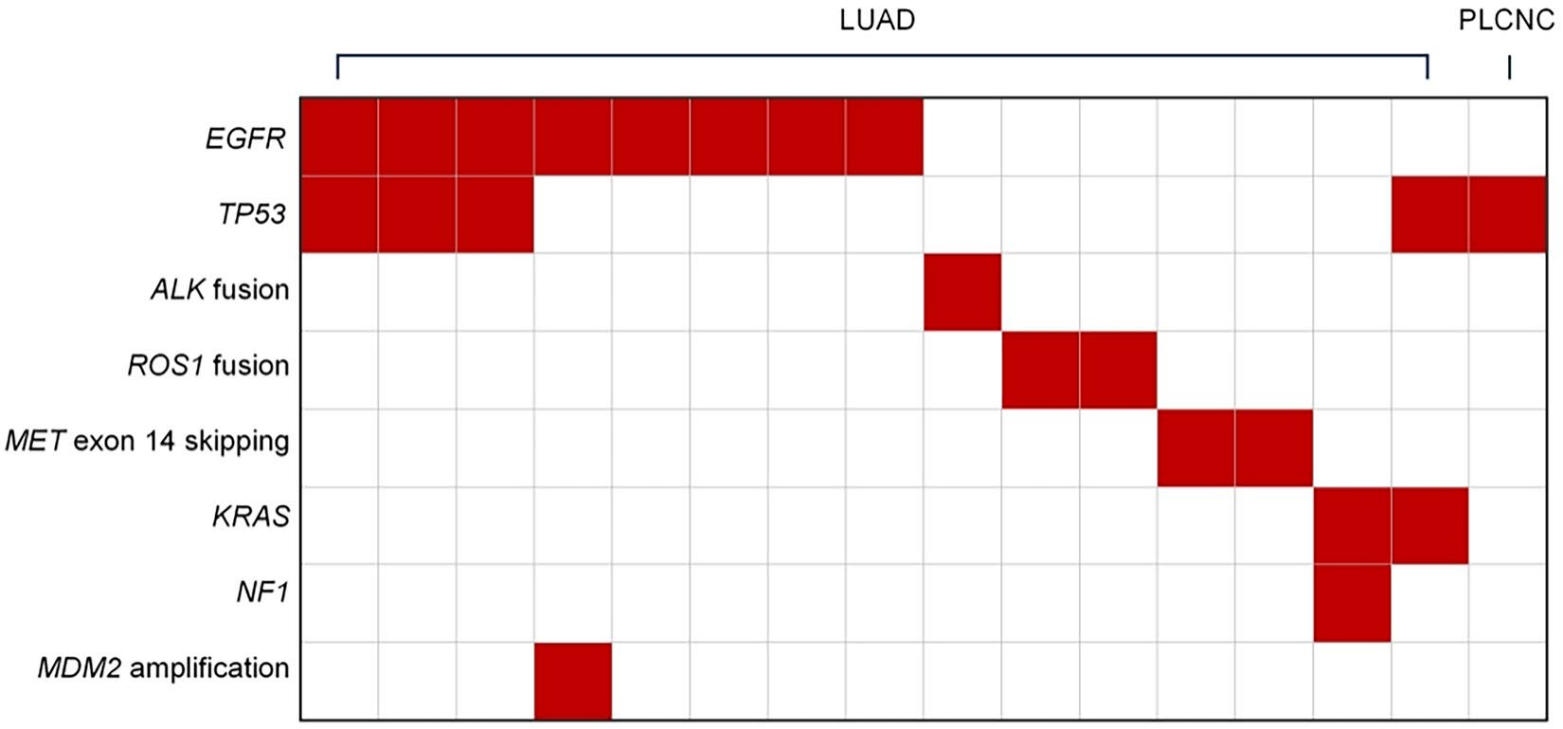
The genetic test results of 16 patients with BE and malignant nodules. LUAD: lung adenocarcinoma; PLCNC: pulmonary large-cell neuroendocrine carcinoma.

Among the 143 patients with pulmonary nodules confirmed by pathology, the pathological findings of benign nodules included inflammation (n = 17, 60.7%), fungal infection (n = 4, 14.3%; comprising aspergillus [n = 3, 10.7%] and cryptococcus [n = 1, 3.6%]), tuberculosis (n = 4, 14.3%), atypical adenomatous hyperplasia (n = 1, 3.6%), hamartoma (n = 1, 3.6%), and hemangioma (n = 1, 3.6%). For malignant nodules, the pathological diagnoses were invasive adenocarcinoma (n = 75, 65.2%), minimally invasive adenocarcinoma (n = 25, 21.7%), squamous cell carcinoma (n = 6, 5.2%), adenocarcinoma *in situ* (n = 5, 4.3%), carcinoid tumor (n = 2, 1.7%), lymphoepithelioma-like carcinoma (n = 1, 0.9%) and pulmonary large cell neuroendocrine carcinoma (n = 1, 0.9%) (Figure 2). Among the 16 patients with malignant nodules who underwent genetic test, the most frequent driver gene mutation was *EGFR* (n = 8, 50%), followed by *TP53* (n = 5, 31.3%). Moreover, targetable driver mutations were identified in 15 patients diagnosed with invasive adenocarcinoma (Figure 3).

For descriptive purposes, we also analyzed all 396 patients with pathological results regardless of clinical data completeness. The radiological features (Table S1) and pathological findings (Figure S1) were broadly consistent with those observed in the main cohort.

**Figure 4.**
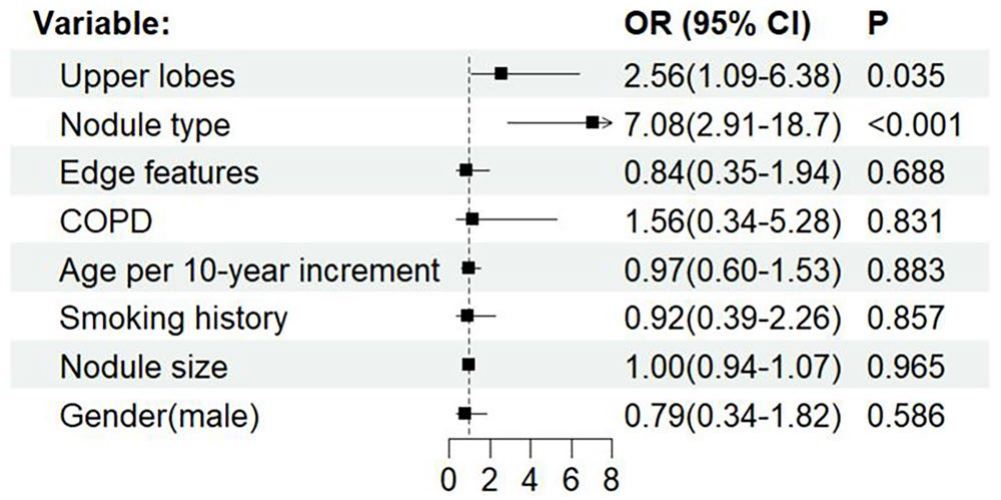
Univariate logistic regression of risk factors for nodule malignancy. Edge features indicate irregular, lobulated, or spiculated edges; Upper lobes indicate nodule located in upper lobes; Nodule type indicates pure ground-glass/ground glass with a solid component nodule type.

Univariate logistic regression analysis showed that location of the nodule in the upper lobe (OR = 2.56, 95%CI: 1.09–6.38, *p* = 0.035) and pure ground-glass/ground glass with a solid component nodule (OR = 7.08; 95%CI: 2.91–18.7; *p* < 0.001) were risk factors for nodule malignancy (Figure 4). Among the seven variables entered into the multivariate model, pure ground-glass/ground glass with a solid component nodule type was the sole independent risk factor for nodule malignancy (adjusted OR = 8.53; 95%CI: 3.14–25.81; *p* < 0.001). Upper lobe location, significant in univariate analysis (OR = 2.56, *p* = 0.035), was attenuated and became non-significant after multivariate adjustment (adjusted OR = 1.93, 95% CI 0.71–5.49, *p* = 0.202) (Table 3).

**Table 3.** Multivariate logistic regression of risk factors for nodule malignancy.

Variable	Multivariate	
	adjusted OR (95% CI)	*P*
Upper lobes*	1.93(0.71–5.49)	0.202
Nodule type**	8.53(3.14–25.81)	<0.001
Edge features***	1.39(0.48–4.13)	0.540
COPD	1.34(0.32–7.22)	0.704
Age per 10-year increment	1.01(0.55–1.78)	0.977
Smoking history	1.05(0.37–3.15)	0.922
Nodule size	1.03(0.95–1.13)	0.438

*Upper lobes indicate nodule location in upper lobe.

**Nodule type indicates pure ground-glass/ground glass with a solid component nodule type.

***Edge features indicate irregular, lobulated, or spiculated edges.

Model includes adjustments for upper lobes, nodule type, edge features, COPD, age per 10-year increment, smoking history and nodule size.

Neither the Reiff score nor the coexistence of BE and pulmonary nodule in the same lobe showed any association with nodule malignancy in univariate logistic regression analysis. Furthermore, after sequential adjustment for multiple potential confounders in multivariable models (including age, gender, smoking history, nodule located in the upper lobe, pure ground-glass/ground glass with a solid component nodule type and edge features), similar results were observed (Table S2).

## Discussion

This study aimed to clarify the clinical, radiological and pathological characteristics of patients with BE and pulmonary nodules, and to identify the risk factor of nodule malignancy. Pure ground-glass/ground glass with a solid component nodule type was identified as an independent predictor of malignancy. Conversely, neither the Reiff score nor the coexistence of BE and pulmonary nodule in the same lung lobe showed a significant associated with nodule malignancy.

Most malignant nodules were located in the upper lobes, presented as pure ground-glass/ground glass with a solid component nodule type and their pathological results were primarily adenocarcinoma. This finding is consistent with previous studies conducted in patients with ground-glass or subsolid pulmonary nodule [9,10]. The upper lobe bronchus is where the largest airflow occurs at the beginning of breath [11,12], therefore the deposition of tobacco smoke particles and the resulting carcinogenic effects are largest in the upper lobe [13,14]. Asian guidelines suggest that high prevalence of tuberculosis may reduce this location’s diagnostic significance for malignancy [15], but only one of the four pulmonary nodules diagnosed with tuberculosis in our study was located in the upper lobe. In univariate analysis, upper lobe location was significantly associated with nodule malignancy (OR = 2.56, *p* = 0.035). However, this association was attenuated and became non-significant in multivariate analysis (adjusted OR = 1.93, 95% CI 0.71–5.49, *p* = 0.202). We attribute this attenuation primarily to confounding by nodule type: upper lobe nodules were more likely to be pure ground-glass/ground glass with a solid component (73.9% of malignant vs. 28.6% of benign nodules, *p* < 0.001), and once nodule type was adjusted for, the independent effect of anatomical location diminished. This suggests that the apparent upper lobe predilection for malignancy in BE patients largely reflects the distribution of histological subtypes rather than representing an independent anatomical risk factor. While upper lobe location was not an independent predictor after multivariate adjustment, the marked difference in prevalence (54.8% vs. 32.1%) suggests it may still serve as a useful contextual indicator when combined with nodule morphology in clinical assessment. Reduced statistical power due to our modest sample size may have also contributed to the widening of confidence intervals. As atypical adenomatous hyperplasia progressed to invasive adenocarcinoma, the nodule transitioned from a pure ground-glass nodule type to a part-solid nodule type [16]. During this transition, *EGFR* and other driver gene mutations played a key role [16]. Our findings were consistent with this model: over half of the malignant nodules presented as pure ground-glass/ground glass with a solid component nodule type, *EGFR* were the most common mutation identified, and all testing results showed at least one known driver mutation.

Previous studies have identified older age, smoking history, larger nodule diameter, emphysema and spiculation as independent predictors of nodule malignancy [17,18]. However, these factors did not emerge as significant predictors in our cohort of patients with BE and pulmonary nodules. We attribute these null findings to three interconnected factors. First, selection bias inherent to our study design: our cohort exclusively comprised patients whose nodules warranted biopsy or surgical resection, representing a pre-selected high-risk population where the discriminatory power of traditional risk factors may be attenuated. Second, limited statistical power: the relatively small sample size (n = 143 total, with only 28 benign cases) undoubtedly reduced our ability to detect modest associations. Third, it is plausible that bronchiectasis-related chronic inflammation modifies the typical risk factor profile, although this hypothesis requires further investigation [19]. Additionally, due to desmoplastic reactions caused by tumor cell infiltration, irregular edges were presented in malignant pulmonary nodules [20]. The predominance of inflammation-related benign nodules (60.7%) in our cohort, many exhibiting spiculated margins due to fibrotic remodeling rather than tumor infiltration, likely further obscured the discriminatory value of morphological features typically associated with malignancy.

In our study, the Reiff score was not associated with nodule malignancy. This is likely because the Reiff score primarily quantifies structure bronchial changes and thus cannot reflect the dynamic inflammatory status [21]. Moreover, the molecular pathogenesis of lung cancer is highly complex and heterogeneous, which arises from a combination of genetic alterations and epigenetic modifications, including gene mutations, amplifications, deletions, and translocations [22]. Accordingly, a structural scoring system such as the Reiff score may be insufficient to capture the inflammatory milieu relevant to lung carcinogenesis. Future studies incorporating biomarkers of systemic or airway inflammation (e.g. IL-6, TNF-α) or disease activity metrics (e.g. exacerbation frequency) may better elucidate this relationship.

A previous study has suggested that pre-existing BE is associated with a reduced risk of lung cancer in the same lobe [19]. However, their study included a small cohort of patients who were already at a higher risk for or more susceptible to lung cancer. The authors hypothesized that elevated serum TGF-β1 levels in BE patients help maintain tissue homeostasis and exert tumor-suppressive effects, although they did not measure serum TGF-β1 in their cohort. In contrast, another study found that BE actually increased the risk of lung cancer [23]. For BE patients, chronic inflammation alone or pulmonary scarring caused by recurrent infection may contribute to lung cancer development [24]. In our study, the coexistence of BE and pulmonary nodule in the same lung lobe was not found to be associated with nodule malignancy. We hypothesized that this may be due to the relatively low Reiff scores and mild structural destruction of BE in the enrolled patients, which indicated that the degree of inflammation was insufficient to cause the pulmonary nodules to become malignant. The different conclusion highlights the complexity of mechanisms underlying pulmonary nodule evolution in the context of BE.

The 52% female proportion in our malignant nodule cohort reflects the predominance of adenocarcinoma (86.9%), a subtype with well-established female predilection in Asian never-smokers, consistent with previous studies in Chinese populations [25–27]. This demographic pattern aligns with Asian guidelines highlighting that women may be more susceptible to lung cancer than men, particularly among never-smokers [15]. The increased risk may be associated with functional genetic differences between the sexes and specific risk factors in Asian women including the widespread use of biomass fuels, passive smoking, cooking smoke and second-hand smoke [27–30].

Previous study showed that hematology-related BE patients exhibited a higher prevalence of *Pseudomonas aeruginosa* isolation and more frequent acute exacerbations, indicating a higher infectious burden [31]. In such immunodeficient individuals who have BE with pulmonary nodules, pulmonary nodules are more likely to infectious-related. However, our study population did not include immunodeficient patients, which means our findings cannot be generalized to immunodeficient patients with BE and pulmonary nodules.

This study has several limitations. First, the high exclusion rate due to missing data (253/474, 53.4%) reflects the challenges of retrospective data collection across heterogeneous clinical settings and may have introduced selection bias. The missing data primarily arose from enrolling patients across multiple departments, and these patients lacked comprehensive documentation of multiple core clinical variables, including smoking history, comorbidities, and spirometry results, etc. Nevertheless, as shown in the (Supplementary Materials Table S1 and Figure S1), the radiological and pathological features of the full pathology‑confirmed cohort were broadly consistent with those of the main clinical analysis cohort, mitigating concerns about severe selection bias for descriptive findings. Second, the restriction to patients with pathological confirmation introduced additional selection bias, inherently excluding low-risk nodules that resolved on follow-up or did not warrant invasive evaluation. Nevertheless, focusing on this subgroup carries immediate clinical utility: it may help avert unnecessary invasive procedures for truly low-risk nodules. Third, the relatively small sample size may have reduced statistical power, potentially obscuring significant associations and precluding more extensive multivariate modeling. Fourth, the lack of available data on BE severity (such as Bronchiectasis Severity Score) and etiological screening results prevented us from a more comprehensive assessment of how chronic inflammation and underlying BE causes might influence lung cancer risk in this population. Finally, as with all single-center retrospective studies, our findings require validation in larger, prospective, multicenter cohorts before broader clinical application.

## Conclusion

Our study investigated the clinical, radiological, and pathological characteristics of BE patients with pulmonary nodule. We identified that pure ground-glass/ground glass with a solid component nodule type was the independent predictor of nodule malignancy. Our findings suggest that nodule morphology, rather than traditional risk factors or bronchiectasis-specific features, may guide malignancy risk assessment in this population.

## Supplementary Material

Supplemental Material

Supplemental Material

## Data Availability

The data that support the findings of this study are available from the corresponding author upon reasonable request.
