# Supplementary material for: Differentiating benign from malignant pulmonary nodules in the context of bronchiectasis: a retrospective study
Source: Ann Med. 2026 Jun 9;58(1):2681234. doi: 10.1080/07853890.2026.2681234 (PMC13250873; doi:10.1080/07853890.2026.2681234)
Supplement: Supplemental Material [file IANN_A_2681234_SM0314.docx]

Supplementary Material

Table S1 Comparison of nodule radiological features between BE patients with benign nodules and malignant nodules

|  | BE with benign nodules  (n=76) | BE with malignant nodules  (n=320) | *P* |
| --- | --- | --- | --- |
| Nodule size, median(IQR) | 16.6(10.6, 20.9) | 17.0(12.2, 21.6) | 0.240 |
| Upper lobes^1^, n(%) | 33(43.4) | 194(60.6) | 0.010 |
| Nodule type  Subsolid nodules  (pGGN^2^/mGGN^3^), n(%)  solid, n(%) | 17(22.4)  59(77.6) | 214(66.9)  106(33.1) | <0.001 |
| Edge features^4^, n(%) | 41(53.9) | 184(57.5) | 0.607 |

^1^Upper lobes mean nodule located in upper lobe

^2^pGGN means pure ground glass nodule, ^3^mGGN means mixed ground glass nodule (ground glass with a solid component nodule)

^4^Edge features mean irregular, lobulated, or spiculated edges

Table S2 Multivariable logistic regression assessing the association of Reiff Score and Bronchiectasis in the same lobe* with pulmonary nodule malignancy

| Model | Reiff score Bronchiectasis in the same lobe* | |
| --- | --- | --- |
|  | OR (95% CI) *P* value | OR(95% CI) *P* value |
| Crude model | 0.91(0.71-1.17) 0.473 | 1.26(0.45-3.22) 0.635 |
| Model 1 | 0.92(0.72-1.18) 0.506 | 1.35(0.50-3.41) 0.541 |
| Model 2 | 0.92(0.72-1.18) 0.513 | 1.36(0.50-3.43) 0.533 |
| Model 3 | 0.99(0.76-1.28) 0.908 | 1.06(0.38-2.75) 0.913 |
| Model 4 | 0.93(0.70-1.23) 0.608 | 0.86(0.29-2.37) 0.781 |
| Model 5 | 0.92(0.69-1.22) 0.561 | 0.92(0.31-2.58) 0.875 |

* Bronchiectasis and pulmonary nodule were located in the same lobe

Model 1 includes adjustments for age and gender

Model 2 is the same as model 1 plus smoking history

Model 3 is the same as model 2 plus nodule location in the upper lobe

Model 4 is the same as model 3 plus pure ground glass/ground glass with a solid component nodule type

Model 5 is the same as model 4 plus edge features

Figure S1. The pathological findings of BE patients with benign nodules and malignant nodules.
