# Supplementary figures and images for: Differentiating benign from malignant pulmonary nodules in the context of bronchiectasis: a retrospective study
Source: Ann Med. 2026 Jun 9;58(1):2681234. doi: 10.1080/07853890.2026.2681234 (PMC13250873; doi:10.1080/07853890.2026.2681234)

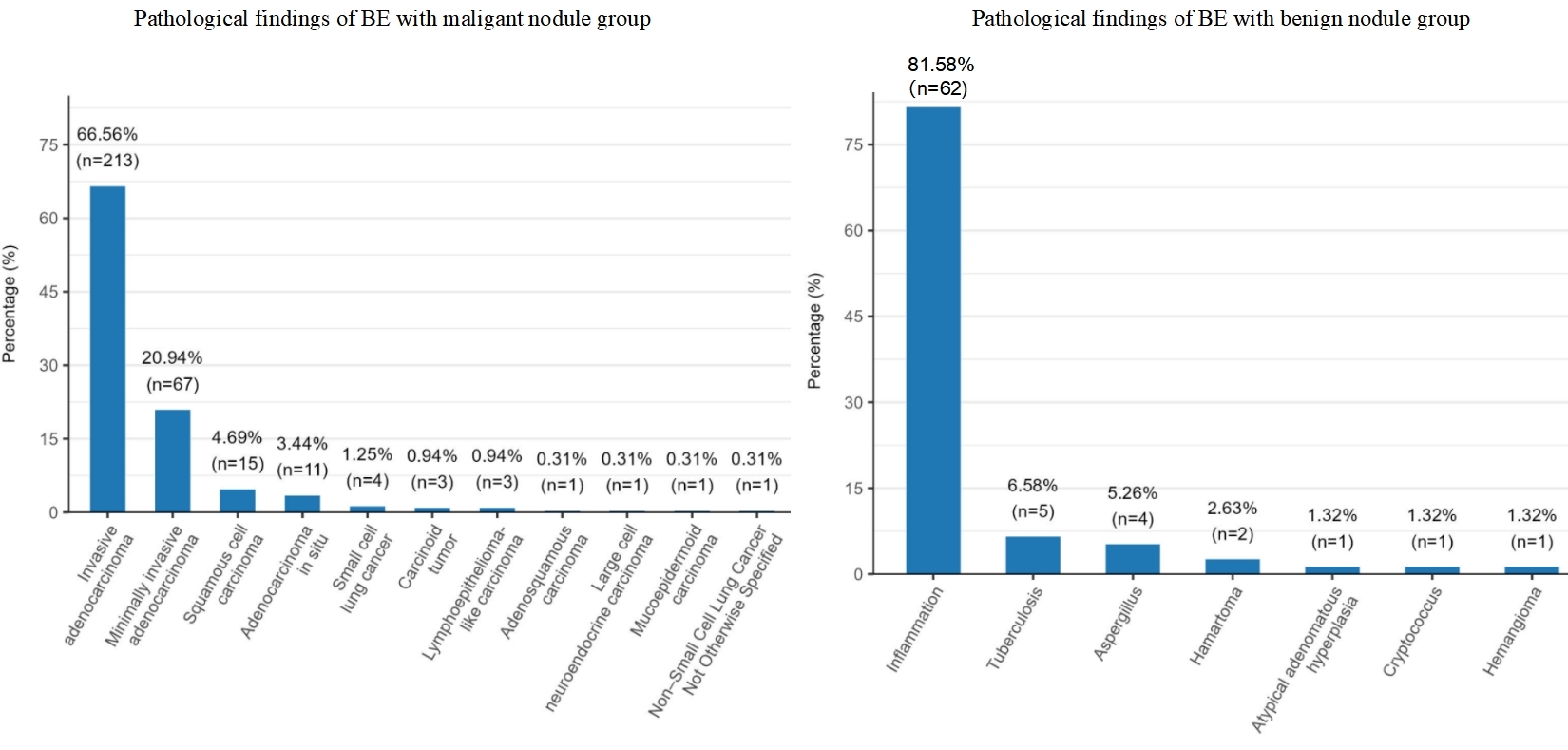

Supplement: Supplemental Material [file IANN_A_2681234_SM5521.jpg]
